# Milk Laboratories

**Published:** 1897-12-25

**Authors:** 


### MILK LABORATORIES.

The attainment of the best methods of rearing infants by hand is generally recognised as a problem of racial importance; but on this side of the Atlantic it is, perhaps, hardly realised how far American physicians have progressed towards its solution. Owing to the enterprise and public spirit of Dr. Rotch, a company has been promoted to carry on, in New York, Boston, and other cities, laboratories in which the production of modified cow's milk is undertaken on a large scale. The laboratories are placed in the charge of highly-skilled officials, who prepare the milk at the prescription of physicians.

Medical men are expected to indicate not only the composition of the food required, but the number of daily meals and the amount of each feeding. They are accustomed to express the proportions of the food required in percentages of the various food-stuffs. For example:—

| | |
|---|---|
| ℞.—Fat ... ... ... ... | 3 per cent. |
| Sugar ... ... ... ... | 6 per cent. |
| Proteid ... ... ... ... | 1 per cent. |
| Alkalinity ... ... ... | 5 per cent. |
| Amount of each meal ... | 4 oz. |
| Number of meals ... ... | 8 |

Heat for 25 minutes at 167 deg. Fahr.

On the receipt of such a prescription, it is dispensed just as any at a druggist's, the ingredients used being separated milk, skimmed cream containing 16 per cent. of fat, milk-sugar, and water. The food supply for an entire day is delivered each morning from the laboratories in glass bottles, closed with cotton-wool plugs; and the bottles are of such a shape that they can be used instead of the usual "infant feeders." The cow's milk used is never sterilised unless it has to be sent long distances, the laboratory experts greatly preferring the process of Pasteurisation by Freeman's apparatus. This process simply involves the maintenance of the temperature of the milk, by means of hot water, at 167 deg. Fahr. for 25 minutes.

This process does not appear to affect, as sterilisation does, the nutritive value of the milk, and, so far, its use has not been followed by rickets or infantile scurvy. The milk, after Pasteurisation, is always kept over ice. In order to guard to the fullest extent against bacterial infection, the company have also become their own cowkeepers.

The dairy farms are administered with scrupulous care; each cow is allotted so many cubic feet of space and the dietary is regulated with scientific precision, Every possible precaution is taken to prevent contamination of the milk during and after collection, and the results show with how great a success.

The establishment of these milk laboratories has been most enthusiastically received by American physicians as a means of safeguarding the life and health of hand-fed infants, and, so far, the results appear to have fully realised the expectations entertained.

One point—the prescription of food in percentages—may seem to offer some little difficulty; but the laborious and excellent work of Dr. Holt has furnished practitioners with guidance on this as on other points. And, after all, one of the great advantages of the system is the opportunity it affords for dieting each case on its individual merits. One or two points in the practice of American physicians are interesting. They employ neither barley nor lime-water as a diluent of the milk, believing that, with the employment of cream rich in fat, the percentage of casein can be safely kept so low that no trouble occurs in its digestion.

Milk-sugar is invariably used in place of cane-sugar, the reasons being that it is less likely to set up gastric fermentation, and that, owing to its lesser sweetening power, it can be used in more generous proportions.

Perhaps we may in time find some of the large dairy companies giving to the inhabitants of London facilities similar to those already possessed by dwellers in Boston and New York. At present the only step in that direction which has been made seems to be the supply of "humanised milk" in two forms, one of which contains more casein than the other. This reduces the "prescribing" to great simplicity, but at the same time does not leave the physician the latitude which he has under the New York system. Moreover, the English "humanised milk" is fully sterilised.

There can be no doubt of the great waste of infant life which occurs in consequence of the devotion of mothers to the fetish of infant foods, and of the uncleanliness of ordinary dairymen. Any system by which pure Pasteurised bottled milk could be supplied at a reasonable price would prevent much disease among infants, while a system such as we have above described would place in the hands of physicians a most efficient, and an almost essential, means of treating those who are ill, and arranging a proper diet for those whose digestion is feeble. The result would certainly be that many lives would be saved to the State, and an enormous amount of infantile suffering prevented. It must be remembered, however, that the great mass of the infantile mortality occurs among the children of the poor, and that the price at which such milk could be dispensed would thus become a matter of great importance.

